# Disposition kinetics of sparfloxacin in healthy, hepatopathic, and nephropathic conditions in chicken after single intravenous administration

**DOI:** 10.4103/0253-7613.55204

**Published:** 2009-06

**Authors:** M.K. Bhar, S. Khargharia, A.K. Chakraborty, T.K. Mandal

**Affiliations:** Department of Pharmacology and Toxicology, West-Bengal University of Animal and Fishery Sciences, 68 KB Sarani, Kolkata-37, India

**Keywords:** Birds, disposition kinetics, healthy, hepatopathic, nephropathic, sparfloxacin

## Abstract

**Objective:**

To study the variation of disposition kinetic values of sparfloxacin in healthy, hepatopathic, and nephropathic chickens after a single intravenous administration.

**Materials and Methods:**

Hepatotoxicity was induced by the administration of paracetamol (500 mg / kg / day, p.o. for seven days) and nephrotoxicity by uranyl nitrate (2.0 mg / kg / day dissolved in distilled water, i.v. for four days) in chickens. Disposition kinetic studies of sparfloxacin were investigated in healthy as well as hepatopathic and nephropathic birds after a single intravenous administration at 40 mg / kg body weight.

**Results:**

Maximum plasma concentration detected at 0.16 hour was 31.25 ± 2.95, 61.95 ±1.85, and 99.86 ± 2.21 μg / ml in healthy, hepatopathic, and nephropathic group, respectively. The drug could not be detected in the plasma of healthy birds beyond 12-hour period, while the same was detectable for 72 hour in the plasma of hepatopathic and nephropathic birds. The concentration of sparfloxacin was significantly (*P* < 0.01) higher in all the samples of hepathopathic and nephropathic birds compared to healthy birds. All the kinetic values were increased (*P* < 0.01) in the hepatopathic and nephropathic birds, except Vd_area_ and Cl_B_ values in hepatopathic Birds; while β and Cl_B_ values nephropathic birds were decreased significantly than that of healthy birds.

**Conclusions:**

The dose of sparfloxacin may be reduced in hepatopathic as well as nephropathic birds.

## Introduction

Sparfloxacin is a potent, long-acting, third generation, bactericidal fluoroquinolone derivative. The drug has shown potent antimicrobial activity against a wide range of Gram positive and Gram negative bacteria, including glucose nonfermentors and anaerobes, *Legionella, Mycoplasma*. *Chlamydia*, and *Mycobacterium* spp. Methicillin-resistant *Staphylococcus aureus* is also susceptible to sparfloxacin.[1] Its absorption through the G.I. tract is slow in human beings due to its lower solubility in aqueous solution. Its elimination half-life ranges from 15 to 22 hours in healthy volunteers, after oral administration.[2] It is well distributed throughout the body with a higher concentration in sinus mucosa, bronchial mucosa, epithelial lining fluid, and alveolar macrophages.[3] It is the drug of choice for treating the lower respiratory tract infection, due to its greater distribution.

Poultry industry in India has developed tremendously in the last two decades. Poultry has enriched human civilization in many ways. Eggs and meat of birds are being consumed since the prehistoric age. Although India has made a major breakthrough in poultry production during the last 25 years, the problems facing this industry are many and diverse. Inadequate health coverage is a major problem. It is also seen that in a number of avian diseases, vital organs like the liver and kidney become affected. Under the conditions, the drugs of choice, route of administration and dosage regimen of the efficient drugs are not clearly known and literature is unavailable that might have dictated the indiscriminate and unscientific use of antibiotics leading to economic loss, and as a consequence poultry birds and poultry farmers suffer a lot.

The kinetic behavior of many drugs was altered following administration by different routes in experimentally-induced diseased models of goats.[4–6] Quinolone derivatives like sparfloxacin are used in poultry to combat the disease caused by susceptible microorganisms. However, literature in respect to pharmacokinetics in healthy and diseased states is scarcely available. The present research work was carried out to study the disposition kinetics of sparfloxacin in healthy birds, its modification in hepatopathic and nephropathic birds, and the data generated thereof to determine the dosage regimen.

## Materials and Methods

### Experimental birds

The study was conducted on birds (42 day old) healthy and induced-hepatopathic and nephropathic conditions, weighing between 1.8-2 kg. The birds were examined clinically to evaluate health status. The animal room was cleaned and fumigated with potassium permanganate and commercial formaldehyde solution (1: 10) 48 hours. The cages, feeding troughs, and watering troughs were cleaned with detergent and disinfected with potassium permanganate solution (5%) 24 hours before starting the experiment. They were housed in cages and were maintained on broiler finisher ration and provided water *ad libitum.*

### Drugs and chemicals

Sparfloxacin in powder form (purity > 80%) was gifted by M/s Alembic Chemicals Works Private Limited, Vadodara. All the chemicals of analytical grade used in the experiment were purchased from E. Merck (India) and Sigma Chemical Company (USA).

### Induction of hepatopathy in birds

Twenty birds were selected for the study and were divided into four groups each consisting of five birds. Birds of group A served as the control, while birds of groups B, C, and D were considered as experimental. Paracetamol suspension in water was administered orally at 250, 500, and 750 mg /kg for seven consecutive days to each bird of groups B, C, and D, respectively. Blood samples were collected on different occasions to measure bromosulfophthalein (BSP) clearance and plasma aspartate aminotransferase activity to assess the intensity of hepatotoxicity. Symptoms of anorexia, dullness, and depression were observed on dosages of 500 and 750 mg/kg. The intensity of hepatotoxicity at different doses of paracetamol was assessed from the BSP clearance plasma aspartate aminotransferase calues and histopathological fin drugs. A minimum dose of 500 mg/kg of paracetamol for seven consecutive days was selected to induce hepatotoxicity.

### Induction of nephropathy in birds

Chemical-induced kidney damage was carried out in birds by the modified method of Datta *et al*.[5] Twenty birds were selected for the study and were divided into four groups of five birds each. Birds from group 1 served as the control, while those from groups 2, 3, and 4 were considered experimental. Uranyl nitrate crystals dissolved in distilled water was administered intravenously to each bird in groups 2, 3, and 4 at 0.75, 2.0, and 4.0 mg/kg, respectively, for four consecutive days. On the fifth day, blood samples were collected from all the birds to measure the level of BUN and creatinine. Symptoms like anorexia and oliguria were recorded at 2 and 4 mg/kg. The intensity of nephrotoxicity induced by intravenous administration of uranyl nitrate at different doses was assessed from the BUN and creatinine levels of blood. Based on the results uranyl nitrate at 2 mg/kg daily for four consecutive days was considered to induce kidney damage.

### Experimental design

Fifteen healthy birds having body weight varying from 1.8 – 2 kg were selected and divided into three groups (Group 1, 2, and 3) of which group 1 was considered as control, while groups 2 and 3 were treated orally with paracetamol at 500 mg/kg and intravenous administration of uranyl nitrate at 2 mg/kg, respectively, to induce hepatopathic and nephropathic conditions.

A single dose of sparfloxacin at 40 mg/kg was administered into the right wing vein of each birds of the healthy (Group I), hepatopathic (Group II), and nephropathic birds (Group III) intravenously.

### Collection of blood

A vein flow catheter (22G) was introduced into the left wing vein of the bird and fixed with adhesive tape. Blood samples were collected through the vein flow catheter of the birds from all the three groups at 0 (predrug control), 0.08, 0.16, 0.25, 0.33, 0.5, 1, 2, 4, 8, 12, 24, 48, and 72 hours post intravenous drug administration, in heparinized test tubes, and centrifuged at 3000 rpm for 30 minutes to separate the plasma for the estimation of the drug.

## Method Validation

### Preparation of calibration curve

A stock solution (100 μg /ml) of sparfloxacin in acetonitrile was prepared. The drug solution was mixed with plasma (0.5 ml) and acetonitrile to make a 5 ml aliquot. Optical densities of the drug molecule of different concentrations (0.5 to 5 μg in 5 ml aliquot) were read at 365 nm, by a double beam UV-VIS spectrophotometer. The concentrations were plotted against optical densities on a graph paper to obtain a standard curve. The recovery percentage of sparfloxacin from the plasma was above 85 and the detection limit for sparfloxacin by UV-VIS spectrophotometer was 1 ppm.

### Estimation of the drug

Plasma (0.5 ml) was mixed with 4.5 ml of acetonitrile to make a volume of 5 ml and shaken for 5 minutes and then centrifuged for 30 minutes at 3000 rpm. The supernatant was collected and read at 365 nm by a double beam UV-VIS spectrophotometer. Concentrations of the drug at different time intervals were obtained from the standard curve prepared previously and expressed as μg /ml of the plasma.

### Pharmacokinetic parameters

Some pharmacokinetic variables like Vd, biological half life, and Cl_B_ were determined using a computerized curve fitting software program “PHARMKIT” supplied by the Department of Pharmacology, Institute of Postgraduate Medical Education and Research, Pondicherry, India. This program was based on a “compartmental model”. The other kinetic parameters were estimated following the standard formula.[10]

### Statistical analysis

The pharmacokinetic parameters for each bird were determined and the mean values and standard error (SE) were calculated. Mean values, SE, analysis of variance, and independent sample ‘t’ test of the tabulated data were calculated, where applicable, using the SPSS statistical software program.

## Results

**Figure 1:**
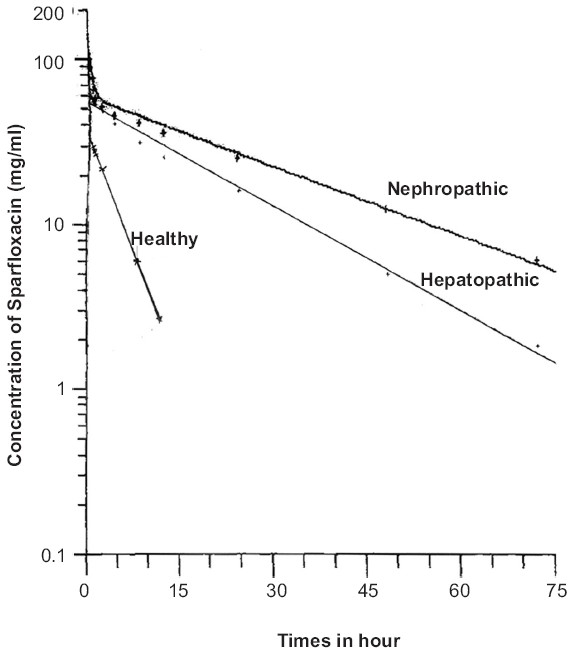
Semilogarithmic plot of mean plasma concentration of sparfloxacin against time with computerized best fit line in healthy, hepatopathic, and nephropathic birds following single dose intravenous administration at 40 mg/kg

A semilogarithmic plot of the mean plasma concentrations of sparfloxacin against time, with a computerized best-fit line, in healthy, hepatopathic, and nephropathic birds after a single-dose intravenous administration at 40 mg/kg have been presented in Figure 1. It has transpired from the figure that maximum plasma concentration of sparfloxacin was 31.25 ± 2.95 μg / ml at 0.16 hour, which was followed by a gradual decline with a minimum plasma drug concentration of 2.55 ± 0.40 μg/ml at 12 hour in healthy birds. On the other hand, C^p^_max_ (maximum plasma concentration) of sparfloxacin was 61.95 ± 1.85 μg /ml at 0.16 hour and C^p^_min_ (minimum plasma concentration) of the drug was recorded to be 1.93 ± 0.19 μg /ml at 72 hour in hepatopathic birds. C^P^_max_ of 99.86 ± 2.21 μg /ml was observed at 0.16 hour, which was followed by a gradual decline in concentration, to attain a C^P^_min_ of 6.13 ± 0.45 μg /ml at 72 hour in nephropathic birds. Sparfloxacin could not be detected in plasma beyond a 12-hour period in healthy and a 72-hour period in hepatopathic and nephropathic birds. It is also evident from Figure 1 that the concentrations of sparfloxacin was significantly (P < 0.01) higher in all the samples of hepatopathic and nephropathic birds compared to healthy birds. The kinetic behavior of sparfloxacin in birds of the healthy and hepatopathic groups followed a “one compartment open model,” while birds of the nephropathic group followed the “two compartment open model” [Figure 1].

The disposition kinetic parameters of sparfloxacin in healthy, hepatopathic, and nephropathic birds following a single-dose intravenous administration at 40 mg/kg have been described in Table 1. It is evident from the table that the values of B, t½ β, AUC, Vd_area,_ and Cl_B_ in healthy birds are 31.45 ± 3.08 μg/ml, 3.40 ± 0.32 hours, 139.77 ± 6.16 μg hours/ml, 0.75 ± 0.08 L /kg, and 0.15 ± 0.005 L/kg/hour, while the same in hepatopathic birds were 53.91 ± 2.48 μg /ml, 14.47 ± 0.35 hours, 1047.97 ± 72.66 μg hour/ml, 0.45 ± 0.03 L/kg, and 0.02 ± 0.002 L /kg/hours The said parameters in nephropathic birds were found to be 57.98 ±1.93 μg /ml, 21.62 ± 0.54 hours, 1587.22 ± 74.50 μg hours/ml, 0.41 ± 0.02 L /kg, and 0.01 ± 0.001 L/kg/hour, respectively. All the kinetic values were significantly altered in hepatopathic and nethropatic birds compared to healthy birds.

**Table 1:** Mean kinetic parameters of sparfloxacin following a single dose intravenous administration at 40 mg/kg in healthy, hepatopathic, and nephropathic birds (Mean of five replicates with SE)

*Kinetic parameters*	*Healthy group*	*Hepatopathic group*	*Nephropathic group*
B (μg /ml)	31.45 ± 3.08	53.91** ± 2.48	57.98* ± 1.93
β (hr^−1^)	0.21 ± 0.02	0.05** ± 0.002	0.03** ± 0.002
1½ β/hr	3.40 ± 0.32	14.47** ± 0.35	21.62** ± 0.54
AUC (μg hr /ml)	139.77 ± 6.16	1047.97** ± 72.66	1587.22** ± 74.50
Vd area (L /kg)	0.75 ± 0.08	0.45* ± 0.03	0.41* ± 0.02
Cl_B_ (L/kg/hr)	0.15 ± 0.005	0.02** ± 0.002	0.01** ± 0.001
MRT (hr)	4.71 ± 0.46	20.69** ± 0.56	31.27** ± 0.77

* *P*<0.05 and

** *P*<0.01 compared to healthy group; β, zero time plasma concentration intercept (elimination phase); β, elimination rate constant; t½ β, biological half-life (elimination phase); AUC, total area under plasma drug concentration versus time curve; Vd_area'_ apparent volume of distribution; Cl_B'_ total body clearance of drug; MRT, mean resident time

## Discussion

The lowest possible detection of sparfloxacin was 1 μg/ml. The total recovery percentages of the drug were found to range from 81.22 to 90.2. The extraction recovery percentages were found to be within the limits and agreeable.

Sparfloxacin persisted for a longer time, with significantly higher concentration (*P* < 0.01) in the plasma of hepatopathic birds compared to healthy birds resulting in significantly higher (*P* < 0.01) AUC value in hepatopathy, and therefore, a significantly decreased value of Vd_area_ was observed in hepatopathic birds. Sparfloxacin undergoes glucuronide conjugation in the liver of rats and humans.[7–9] A metabolism study of sparfloxacin was not carried out in the present experiment and reports on metabolism in birds are scarcely available. It is expected that metabolism of sparfloxacin as well as excretion through the biliary duct may be interfered with, due to hepatic damage resulting in a significantly higher concentration and longer persistence of sparfloxacin in birds.

Concentration of sparfloxacin was also significantly higher (*P* < 0.01) in nephropathic birds compared to healthy birds leading to higher t_1/2β_ and AUC values, and a lower Vd_area_ value. Lesser amount of unchanged sparfloxacin is excreted through the urine of healthy humans[8] and a major amount undergoes hepatic clearance, which includes metabolism and biliary excretion. Baggot[10] reported that uremia decreases the metabolism of the drug in the liver. Most of the fluoroquinolones are eliminated primarily through glomerular filtration and tubular secretion in the kidney.[11] Shimada *et al*.[1] suggested that the excretion of sparfloxacin is predominantly through glomerular filtration instead of tubular secretion, therefore, the decreased value of Cl_B_ of sparfloxacin in acute renal failure in birds might be due to reduced glomerular filtration as evidenced in the uremic condition. From the present research work it may be concluded that the dose of sparfloxacin should be reduced in hepatopathic as well as nephropathic birds.
